# Hydroxylpropyl-β-cyclodextrin as Potential Excipient to Prevent Stress-Induced Aggregation in Liquid Protein Formulations

**DOI:** 10.3390/molecules27165094

**Published:** 2022-08-10

**Authors:** Tanja Stolzke, Franziska Krieg, Tao Peng, Hailong Zhang, Olaf Häusler, Christoph Brandenbusch

**Affiliations:** 1 Laboratory of Thermodynamics, Department of Biochemical and Chemical Engineering, TU Dortmund University, 44227 Dortmund, Germany; 2 Roquette Asia Pacific Pte. Ltd., Singapore 138588, Singapore; 3 Roquette Freres, 62080 Lestrem, France

**Keywords:** 2-hydroxylpropyl-β-cyclodextrin, Kleptose^®^HPB, auto-association, monoclonal antibody, protein stability, surface tension, molecular interactions, formulation design, IgG, adalimumab

## Abstract

Due to the growing demand for patient-friendly subcutaneous dosage forms, the ability to increasing protein solubility and stability in formulations to deliver on the required high protein concentrations is crucial. A common approach to ensure protein solubility and stability in high concentration protein formulations is the addition of excipients such as sugars, amino acids, surfactants, approved by the Food and Drug Administration. In a best-case scenario, these excipients fulfil multiple demands simultaneously, such as increasing long-term stability of the formulation, reducing protein adsorption on surfaces/interfaces, and stabilizing the protein against thermal or mechanical stress. 2-Hydroxylpropyl-β-cyclodextrin (derivative of β-cyclodextrin) holds this potential, but has not yet been sufficiently investigated for use in protein formulations. Within this work, we have systematically investigated the relevant molecular interactions to identify the potential of Kleptose^®^HPB (2-hydroxylpropyl-β-cyclodextrin from Roquette Freres, Lestrem, France) as “multirole” excipient within liquid protein formulations. Based on our results three factors determine the influence of Kleptose^®^HPB on protein formulation stability: (1) concentration of Kleptose^®^HPB, (2) protein type and protein concentration, and (3) quality of the protein formulation. Our results not only contribute to the understanding of the relevant interactions but also enable the target-oriented use of Kleptose^®^HPB within formulation design.

## 1. Introduction

With sales of approximately USD 127 billion in 2021, monoclonal antibodies (mAbs) represent the dominant drug type within the biopharmaceutical sector [1]. Historically, due to their low solubility in water, they were/are frequently formulated at lower concentrations and administered intravenously by medical professionals. However, in recent years, the trend shifted toward subcutaneous (SC) (self) administration, with the mAb being formulated at higher concentrations (>100 mg/mL), yielding a low volume for injection. Between 2008 and 2017, approximately 45 mAb-based products have been approved by the Food and Drug Administration (FDA). About 16 (35%) of those allow for a subcutaneous administration [2]. Although SC formulations show higher patient compliance and acceptance as they can be administered at home by the patient itself [2,3], the design of SC formulations is challenging. Besides mAb solubility (>100 mg mL^−1^) and stability at these high concentrations, also solution viscosity (to allow for syringe ability) has to be taken into account. Furthermore, as SC formulations are no longer administered in a “protected, clinical environment”, incorrect handling by the patient (e.g., shaking of syringes/formulations, or exposure to different temperatures) are likely to result in an increased risk of aggregation and physical instability of the formulation.

One approach to tackle those challenges, and meet the requirements for SC administered high-concentration protein formulations (HCPFs), is the addition of excipients of various classes (such as sugars, amino acids, surfactants, polymers, antioxidants etc.,) to solubilize and stabilize the mAb in solution. Besides classical excipients that are approved by the FDA, a growing demand for novel and innovative (“multirole”) excipients has emerged. These excipients fulfill multiple demands simultaneously, such as (1) increase long-term stability, (2) reduce protein adsorption to surfaces/interfaces, (3) reduce the interfacial tension, and (4) prevent aggregation by mechanical or thermal stress induced e.g., by mishandling of the formulation.

**Figure 1:**
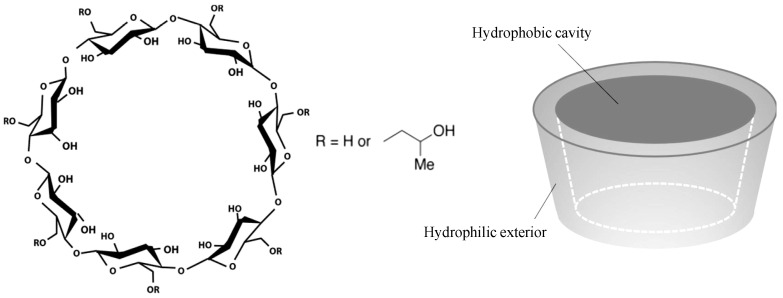
Schematic chemical structure and conformation of 2-hydroxypropyl β-cyclodextrin (HP-β-CD) [17].

An example of these emerging modalities is 2-hydroxypropyl β-cyclodextrin (HP-β-CD) (see Figure 1), which is a promising derivative of β-CD. Both β-CD and especially HP-β-CD show some surface activities (similar to surfactants) and act as stabilizers due to their sugar-based structure (like polyols) (see Figure 1). The truncated cone-like conformation of the glycopyranose units with the orientations of hydroxyl functions and primary/secondary hydroxyl groups of glucose residues lead to their hydrophilic exterior and hydrophobic cavity behavior [4,5,6]. These properties favor the formation of inclusion complexes, allowing β-CD and the derivatives (used as complexing agents) to improve the solubility, stability, and the drug delivery of complex (small)-molecule pharmaceuticals in almost any drug formulation type (oral, IV, SC etc.,) [7,8]. With an improved stability and solubility in water (≤600 mg mL^−1^ [9]) and additional lower toxicity HP-β-CD is a more potential excipient compared to β-CD.

Since its discovery about 30 years ago [10,11], HP-β-CD is especially known in pharmaceutical industry in the field of small-molecule pharmaceuticals to ensure the solubility of the active pharmaceutical ingredients (API) and the stability of the formulation (e.g., in amorphous solid dispersions). It further enhances the bioavailability and dissolution behavior of API [9,12,13,14,15]. However, HP-β-CD is only rarely applied to (liquid) protein formulations. About 35 patents exist describing IgG-based formulations containing HP-β-CD [16] with an even lower number on actual publications on IgG-based formulations containing HP-β-CD (~10) [16].

According to Wu et al. [16] and Serno et al. [18], two main (stabilization-) mechanisms are known for HP-β-CD with respect to HCPF design.

(i) HP-β-CD is expected to reduce the aggregation propensity of the protein due to a direct interaction/attachment with the protein. This hypothesis was later confirmed by Samra et al. [19] for three different proteins. Härtl et al. [20] further confirmed this stabilization effects (for two different immunoglobulin G type antibodies) by means of static and dynamic light scattering analytics (SLS/DLS—determination of second osmotic virial coefficient *B*_22_ or diffusion interaction parameter *k_D_*). According to their results, the interaction between HP-β-CD and IgG reduces the apparent hydrophobicity of the protein and thus decreases the adsorption tendency (increase in *B*_22_-values with increasing HP-β-CD concentration, indicating rise in net repulsive protein-protein interactions).
(ii) HP-β-CD is expected to reduce the surface tension at the liquid/air interface resulting in a decrease in interface-related instability of IgG (in HCPF). Studies by Sá Couto et al. [21] and Loftsson [22] have already shown a decrease in surface tension due to the presence of HP-β-CD in aqueous solutions. However, the potential of HP-β-CD serving as a surfactant agent and thus stabilizer within HCPF is limited, as shown by the studies according to Serno et al. [18,23,24]. Serno et al. [24] investigated the surface activities of HPB and PS80 by dynamic surface tension analysis and drop profile analysis and identified a lower influence of HP-β-CD (~68 mN m^−1^—0.35% *w*/*v*) on the surface tension than for PS80 (~52 mN m^−1^—0.004% *w*/*v*). Thus, HP-β-CD cannot reduce the IgG aggregation at the liquid/air interface to the same extent as other non-ionic surfactants [24].

In general, available data shows that the influence on the surface activity, as well as interactions between HP-β-CD and IgG within the formulations have to be taken into account to evaluate HP-β-CD as “multi-role” excipient within HCPFs.

Within this work, according to the guidelines of Wu et al. [16], we investigate the relevant molecular interactions systematically using established methods to identify the potential of Kleptose^®^HPB (Roquette Freres, Lestrem, France) as excipient within liquid protein formulations (that is HCPFs). We determined:

- The aggregation propensity as function of Kleptose^®^HPB (Roquette) in the absence and presence of a model mAb, that is γ-globulins from human blood (HB-IgG-A) [hydrodynamic radius *r_h_* using dynamic light scattering method (DLS)],
- The concentration-dependent phase behavior of Kleptose^®^HPB in formulations containing HB-IgG-A (surface tension using pendant drop method),
- The individual influence of Kleptose^®^HPB on (pre-existing) mAb aggregates, monomers and fragments [mass fractions within formulations using combination of size-exclusion chromatography and multi-angle light scattering (SEC-MALS)],
- The influence of Kleptose^®^HPB on the conformational and colloidal stability of the two different proteins HB-IgG-A and adalimumab (unfolding temperature *T_unfold_* and second osmotic virial coefficient *B*_22_ using nano differential scanning fluorimetry (nanoDSF) and static light scattering method (SLS), respectively).

Furthermore, we investigated the influence of Kleptose^®^HPB on the stability of HB-IgG-A and adalimumab under both storage (4 °C) and accelerated, stressed (40 °C) conditions by means of aggregation temperature *T_agg_*, melting temperature *T_m_* and aggregate fraction of the respective protein. Based on these results obtained, we provide a holistic conclusion on the potential of Kleptose^®^HPB as a “multi-role” excipient within HCPFs.

## 2. Materials and Methods

### 2.1. Materials

The chemicals used within this work are listed in Table 1. The masses of components were weighed in with a BP 201S scale (Sartorius, Göttingen, Germany) with an accuracy of ±0.1 mg. To assess protein aggregation in the presence of Kleptose^®^HPB, HB-IgG from two different commercial sources (Type A and Type B) was used without further purification.

### 2.2. Methods

#### 2.2.1. Dynamic Light Scattering

The hydrodynamic radius $r_{h}$ of particles ($r_{h}$ > 0.2 nm) within solutions can be determined according to Stokes–Einstein equation [25] [see Equation (1)]:

(1) $$r_{h}=\frac{k_{B}\cdot T}{6\cdot \pi \cdot \eta \cdot D_{t}}$$

$k_{B}$ is the Boltzmann constant, T is the temperature, and η is the viscosity of the solution. The translational diffusion coefficient $D_{t}$ results from the analysis of the autocorrelation function gained from signal fluctuation (fluctuation of light scattering due to Brownian molecular motion [25]). To determine $r_{h}$ of particles within aqueous Kleptose^®^HPB solution in absence and presence of HB-IgG-A (η-data required—see Table A2 in the Appendix A of the manuscript), $D_{t}$ was measured as a function of Kleptose^®^HPB concentrations using dynamic light scattering device DynaPro^®^ NanoStar^®^ from Wyatt Technology Corporation (Santa Barbara, CA, USA). Due to two photodiodes (1st with a correlator for the analysis of DLS; 2nd arranged at a 90° angle for the analysis of the static light scattering (SLS) of the sample), the hydrodynamic radius $r_{h}$ ($r_{h}$ > 0.2 nm [26]) and the weight average molecular weight $M_{W}$ were determined at the same time. The software DYNAMICS^®^ Version 7.9.0.5 from Wyatt Technology Corporation (Santa Barbara, CA, USA) was used to determine $r_{h}$ and $M_{W}$ from the scattering intensities of the online measurement. The laser wavelength is 656 nm.

All solutions investigated are based on a 50 mM K_2_HPO_4_-NaH_2_PO_4_ buffer solution at pH 7 until otherwise stated. Kleptose^®^HPB was first dissolved in buffer solution and, if necessary, the pH was adjusted with 1 M NaOH/1 M HCl. For solution containing HB-IgG-A, the lyophilized protein is completely dissolved in the Kleptose^®^HPB buffer solution prepared previously and the pH is checked/adjusted. The solutions were then centrifuged for 15 min at 3900 rpm and 25 °C using a Centrifuge 5810 R (Eppendorf, Hamburg, Germany).

The duplicate measurements were done for filtered (0.2 µm polyethersulfone syringe filter, Whatman, Buckinghamshire, UK) and non-filtered samples in lean buffer solution (eight Kleptose^®^HPB concentrations) and HB-IgG-A solution with concentration of 30 mg mL^−1^ (three different Kleptose^®^HPB concentrations) at 25 °C and pH 7. The quartz cell JC-265 from Wyatt Technologies Corporation (Santa Barbara, CA, USA) was filled with 150 µL of the respective aqueous solution.

#### 2.2.2. Pendant Drop

The surface tension σ of liquid protein formulations in presence of different excipients (here: Kleptose^®^HPB) can be determined according to the combined relation of Young–Laplace equation [27] and hydrostatic law (see Equation (2)):

(2) $$\sigma \cdot \left(\frac{1}{R_{1}}+\frac{1}{R_{2}}\right)=\Delta \rho \cdot g\cdot z$$

Here, $R_{1}$ and $R_{2}$ are the radii of curvature/curved liquid surface (drop formed), Δρ denotes the difference in density between the inner and outer medium of the drop, 𝑔 is the gravitational force and 𝑧 describes the length of drop (z-coordinate). In order to determine σ of aqueous HB-IgG-A solution as function of Kleptose^®^HPB concentration, the shape of a pendant drop is measured using the OCA 15E tensiometer from dataphysics (Filderstadt, Germany).

For the investigation, Kleptose^®^HPB and HB-IgG-A (lyophilized) were dissolved in 50 mM K_2_HPO_4_-NaH_2_PO_4_ buffer solution at pH 7. The solutions were then centrifuged for 15 min at 3900 rpm and 25 °C using a Centrifuge 5810 R (Eppendorf, Hamburg, Germany). All samples were filtered with a polyethersulfone (PES) syringe filter from VWR International GmbH (Darmstadt, Germany) with a pore size of 0.45 μm.

For determination of σ, a drop of the solution prepared was formed within the measuring cell using a 100 μL syringe (Hamilton Company), through a cannula with an outer diameter of 0.31 mm. The drop shape was analyzed and σ was determined (based on reference—water drop at 25 °C with σ = 71.97 mN m^−1^ [28]) using the SCA software from dataphysics. The duplicate measurements were performed with solutions containing 30 mg mL^−1^ HB-IgG-A and Kleptose^®^HPB (two different concentrations) at 25 °C.

#### 2.2.3. Nano Differential Scanning Fluorimetry

The unfolding, melting, and aggregation temperature of proteins ($T_{unfold}$, $T_{m}$, and $T_{agg}$, respectively) can be determined simultaneously by using the Prometheus NT 0.48 from NanoTemper (Munich, Germany), due to combined measurement principles of nano differential fluorimetry and light scattering [29,30,31,32,33,34].

In order to determine $T_{unfold}$ and $T_{m}$ of the protein, the heat-induced change in the intrinsic dual UV-fluorescence of tryptophan and tyrosine (due to unfolding) is measured at the wavelengths λ=330 nm and λ=350 nm using Prometheus NT 0.48 [35,36]. $T_{unfold}$-value is obtained from onset point and $T_{m}$-value is given by the inflection point of the unfolding profile which is gained by plotting the ratio of fluorescence (F350/F330) against temperature [33,37,38]. $T_{agg}$ is determined by measuring the inverse light scattering (also known as back-reflection technology—NanoTemper, Munich, Germany) as function of temperature. The plot of inverse light scattering signal over the temperature provides the aggregation profile while $T_{agg}$-value results from temperature/initial point of increased inverse light scattering signal [34,39,40,41].

Both principles of operation are carried out in parallel, so the determination of $T_{unfold}$, $T_{m}$, and $T_{agg}$ of the protein is performed simultaneously.

Kleptose^®^HPB were first dissolved in the 50 mM K_2_HPO_4_-NaH_2_PO_4_ buffer solution at pH 7 (containing HB-IgG-A) or pH 5.2 (containing adalimumab). If necessary, the pH was adjusted with 1 M NaOH/1 M HCl. For samples containing HB-IgG-A, the lyophilized protein is completely dissolved in the Kleptose^®^HPB buffer solution and the pH is checked/adjusted. The solutions are then centrifuged for 15 min at 3900 rpm and 25 °C using a Centrifuge 5810 R (Eppendorf, Hamburg, Germany) and filtered with a PES syringe filter with a pore size of 0.45 μm (VWR International GmbH, Darmstadt, Germany). Samples containing adalimumab are based on a protein solution provided by Roquette Asia Pacific Pte. Ltd. (Singapore), which has been depleted of excipients and transferred to 50 mM K_2_HPO_4_-NaH_2_PO_4_ buffer solution at pH 5.2 (using Vivaspin Turbo 15 tubes with 30,000 molecular weight cut-off (Sartorius, Göttingen, Germany) and Centrifuge 5810 R (Eppendorf, Hamburg, Germany) with 3900 rpm at 5 °C). The retentate (adalimumab concentration of ~15 mg mL^−1^) was sterile filtered with a polyethersulfone (PES) syringe filter with a pore size of 0.2 µm (Whatman, Buckinghamshire, UK). Kleptose^®^HPB is dissolved in 50 mM K_2_HPO_4_-NaH_2_PO_4_ buffer solution at pH 5.2 and subsequently added to the adalimumab solution (adalimumab concentration of ~10 mg mL^−1^).

The duplicate measurements were performed with HB-IgG-A solution containing Kleptose^®^HPB (six different concentrations) as well as with adalimumab solution containing Kleptose^®^HPB (two different concentrations). About 10 µL of the aqueous HB-IgG-A solution ($c_{HB-IgG}$ = 30 mg mL^−1^) or adalimumab solution ($c_{adalimumab}$ = 10 g L^−1^) were filled into glass capillaries. The measurements were performed with a heating rate of 0.5 K min^−1^ from 20 °C to 85 °C.

#### 2.2.4. Composition-Gradient Multi-Angle Light Scattering

In order to determine the protein–protein interactions influenced by different excipients (here: Kleptose^®^HPB) the second osmotic virial coefficient $B_{22}$ of proteins (here: HB-IgG-A or adalimumab) can be determined according to the following Equation (3) [42]:

(3) $$\frac{K_{SLS}\cdot {\left(\frac{dn}{dc_{protein}}\right)}^{2}\cdot c_{protein}}{\Delta R\left(c_{protein}\right)}=\frac{1}{M_{protein}}+2\cdot B_{22}\cdot c_{protein}$$

Here, $K_{SLS}$ is the optical constant of the apparatus, $dn/dc_{protein}$ accounts to the refractive index increment (within this work for HB-IgG-A calculated via the Clausius-Mossotti equation, and for adalimumab assumed to be 0.185), $\Delta R\left(c_{protein}\right)$ is the excess Rayleigh ratio, $c_{protein}$ and $M_{protein}$ are the concentration and the weight average molecular weight of the corresponding protein (HB-IgG-A or adalimumab). Similar to previous publications of our research group [43,44,45], for experimentally determining the $B_{22}$-values of protein (via a Zimm Plot; e.g., see [44]), $\Delta R\left(c_{HB-IgG}\right)$ was measured for different scattering angles and protein concentrations in aqueous solution (same sample preparation as described in previous Section 2.2.3) using composition gradient multi-angle light scattering (CG-MALS). The setup consists of a pump and dosing unit (Calypso^®^ II), a MALS unit (DAWN HELEOS 8+, laser wavelength of 664.5 nm) which features a flow-through cell consisting of quartz glass and eight detectors arranged in a semi-circle around the sample and a RI detector (Optilab^®^ T-rEX, laser wavelength of 658 nm) all from Wyatt Technology Corporation (Santa Barbara, CA, USA). The light scattering signals are analyzed and $B_{22}$ is determined using the software ASTRA^®^ V.7.3.2 (Wyatt Technology Corporation, Santa Barbara, CA, USA). The measurements were performed at 25 °C and pH 7 (HB-IgG-A) or pH 5.2 (adalimumab) in duplicate. $B_{22}$-measurements were performed for HB-IgG-A or adalimumab solutions containing Kleptose^®^HPB (six or two different concentrations, respectively).

#### 2.2.5. Combination of Size-Exclusion Chromatography and Multi-Angle Light Scattering

The aggregation of components (here: Kleptose^®^HPB, HB-IgG-A, HB-IgG-B) within aqueous formulations can be determined by aggregate fraction using size-exclusion chromatography. The sample is fractionated according to size and the aggregate fraction, which has a shorter retention time (earlier elution) due to its larger size, can be determined. The SEC can be combined with MALS to record the sample separation qualitatively or quantitatively by detectors.

As described in our previous study [45], the SEC-MALS setup used consists of Q1260 Infinity II Quarternary system from Agilent Technologies (Santa Clara, CA, USA) including a pumping and degassing unit (G7111B), an auto sampler (G7129A), a SEC column (Superdex 200 Increase 10/300 GL) from Cytiva (Marlborough, MA, USA), a diode array detector (G7115A), a RI-detector (G7162A), and a light scattering detector (miniDAWN, Wyatt Technology Corporation, Santa Barbara, CA, USA). The detection with the diode array detector was performed at a wavelength of 280 nm.

The samples were prepared as follows: Dissolution of Kleptose^®^HPB in 50 mM K_2_HPO_4_-NaH_2_PO_4_ buffer solution at pH 7; if HB-IgG-A is also presented within the formulation, HB-IgG-A (lyophilized) is dissolved within the aqueous Kleptose^®^HPB buffer solution; Centrifugation for 15 min at 3900 rpm and 25 °C using a Centrifuge 5810 R (Eppendorf, Hamburg, Germany); Filtration using PES syringe filter from Whatman (Buckinghamshire, United Kingdom) with a pore size of 0.2 μm. The duplicate measurements were performed for solutions containing Kleptose^®^HPB (two different concentrations) in presence of HB-IgG-A (0.8 g L^−1^). Samples containing HB-IgG-B were prepared in the same way as described for HB-IgG-A samples and used for the experiments.

The duplicate measurements of long-term samples (sample preparation as described in Section 2.2.4) were performed with a protein concentration of 1 g L^−1^.

A 50 mM K_2_HPO_4_-NaH_2_PO_4_ buffer solution at pH 7 was used as the mobile phase. The flow rate was chosen to 0.75 mL min^−1^ combined with a sample injection volume of 100 µL over 35 min (method duration) at 25 °C. The ASTRA^®^ V.7.3.2 software from Wyatt Technology Corporation (Santa Barbara, CA, USA) was used to evaluate the respective mass fraction (aggregate, monomer, and fragment) and weight average molecular weight within the sample.

The assessment of HB-IgG-B aggregation and fragmentation was conducted on a Waters high resolution SEC column (XBridge Premier Protein SEC 250 Å, 2.5 μm, 7.8 × 300 mm). All the runs were carried out in 50 mM K_2_HPO_4_-NaH_2_PO_4_ buffer solution with 150 mM NaCl at pH 7. The data were collected and analyzed by Waters Empower^TM^ 3.0 Software (Milford, MA, USA). All sample preparation and other HPLC conditions were same as for HB-IgG-A studies.

#### 2.2.6. Long-Term Samples and Storage Conditions

Samples needed for long-term stability tests were prepared with HB-IgG-A or adalimumab (Roquette Asia Pacific Pte. Ltd., Singapore) and were stored at both 4 °C (over at least 24 weeks in a fridge) and 40 °C (stressed, accelerated stability studies over at least 12 weeks in a ThermoMixer from Eppendorf, Hamburg, Germany). The choice of storage temperatures and duration are in good agreement with FDA guidance and common practice [46,47,48].

HB-IgG-A (lyophilized) was dissolved in 50 mM K_2_HPO_4_-NaH_2_PO_4_ buffer solution (pH 7) with resulting concentration of 95 mg mL^−1^. The excipients or excipient mixtures (Kleptose^®^HPB, l-arginine or trehalose) were dissolved in 50 mM K_2_HPO_4_-NaH_2_PO_4_ buffer solution. The pH of the solution was adjusted if necessary (1 M NaOH/1 M HCl) and the solutions were filtered using 0.45 µm pore size polyethersulfone (PES) syringe filters. The HB-IgG-A- and excipient solutions were mixed with respect to desired excipient concentration (total volume of 1.7 mL with 85 mg mL^−1^ HB-IgG-A).

Samples containing adalimumab are based on a protein solution provided by Roquette Asia Pacific Pte. Ltd. (Singapore), which has been depleted of excipients and transferred to 50 mM K_2_HPO_4_-NaH_2_PO_4_ buffer solution at pH 5.2 (using Vivaspin Turbo 15 tubes with 30,000 molecular weight cut-off (Sartorius, Göttingen, Germany) and Centrifuge 5810 R (Eppendorf, Hamburg, Germany) with 5000 rpm at 5 °C). The retentate (adalimumab concentration of ~25 mg mL^−1^) was sterile filtered with a polyethersulfone (PES) syringe filter with a pore size of 0.2 µm (Whatman, Buckinghamshire, UK). The preparation of excipients or excipient mixtures (Kleptose^®^HPB, l-arginine or trehalose) solution is similar to that described above, and differs only in the solution pH of 5.2. The adalimumab- and excipient solutions were mixed resulting in adalimumab concentration of ~20 mg mL^−1^.

## 3. Results

### 3.1. Hydrodynamic Radii

**Figure 2:**
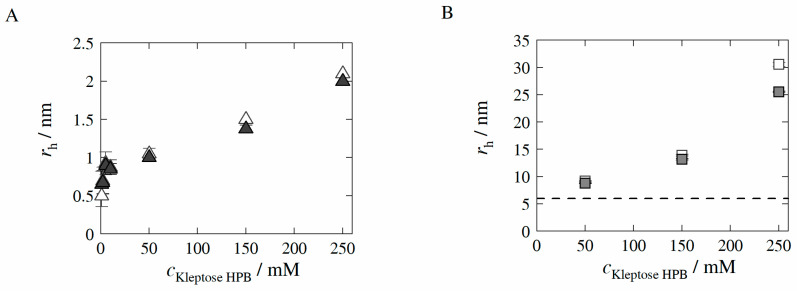
Experimentally determined hydrodynamic radii *r_h_* in the absence of HB-IgG-A (**A**) and presence of HB-IgG-A (30 mg mL^−1^) (**B**) as a function of Kleptose^®^HPB concentration in 50 mM K_2_HPO_4_-NaH_2_PO_4_-solution (pH 7) at 25 °C. Blank symbols illustrate resulting *r_h_* of unfiltered samples, whereas filled symbols illustrate resulting *r_h_* of filtered samples (PES syringe filter, pore size of 0.2 µm). The black dashed line indicates *r_h_* of model protein HB-IgG-A in 50 mM K_2_HPO_4_-NaH_2_PO_4_-solution (30 mg mL^−1^ and pH 7).

In order to identify the aggregation propensity of Kleptose^®^HPB in absence (auto-association) as well as in presence of model protein HB-IgG-A, the hydrodynamic radii $r_{h}$ of possible particle/aggregate formation were determined experimentally by means of dynamic light scattering (see Section 2.2.1). All samples were measured unfiltered, and filtered using a PES syringe filter (pore size: 0.2 µm). Experimentally determined $r_{h}$-values are illustrated in Figure 2 for aqueous Kleptose^®^HPB solution (A) and Kleptose^®^HPB—HB-IgG-A solutions (B) as a function of Kleptose^®^HPB concentration.

**Figure A1:**
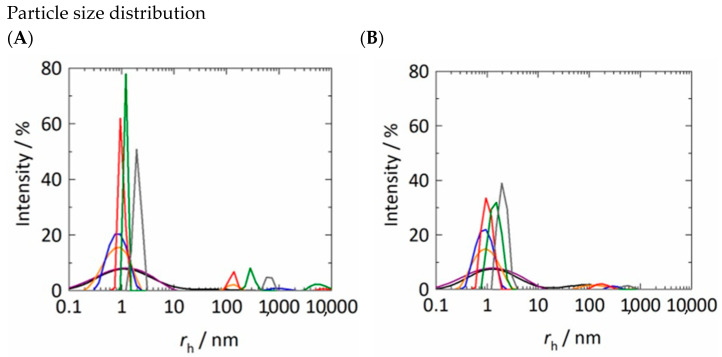
Experimentally determined particle size distributions of unfiltered (**A**) and filtered (**B**) (PES syringe filter with a pore size of 0.2 μm) samples as function of Kleptose^®^HPB in 50 mM K_2_HPO_4_-NaH_2_PO_4_ solution at 25 °C. The different colored lines indicate the different concentrations of Kleptose^®^HPB (1 mM—black; 2 mM—purple; 5 mM—orange; 10 mM—blue; 50 mM—red; 150 mM—green; 250 mM—gray).

The results reveal that $r_{h}$-values of both unfiltered (blank triangles in Figure 2A) and filtered (filled triangles in Figure 2A) samples increase with increasing Kleptose^®^HPB concentration, indicating increasing aggregate size (auto-association) of Kleptose^®^HPB. The slope of the increase in $r_{h}$-values is higher for low Kleptose^®^HPB concentrations (1 to 10 mM) compared to high Kleptose^®^HPB concentrations (10 to 250 mM), revealing a sharp increase in auto-association following the onset at low Kleptose^®^HPB concentrations (see Figure 2A). Furthermore, $r_{h}$-values of the unfiltered and filtered samples ($c_{Kleptose^{®}HPB}$ > 50 mM) reveal (see difference blank and filled triangles in Figure 2A and particle size distribution in Figure A1 given in the Appendix A of the manuscript), the formation of small < 2.2 nm aggregates and some significantly larger aggregate assemblies (>100 nm). The decrease in $M_{w}$-values (weight average molecular weight) of the filtered Kleptose^®^HPB samples with Kleptose^®^HPB concentrations higher than 50 mM (see Table A3 given in the Appendix A of the manuscript) confirms that these larger aggregates are retained by the syringe filter.

If the target mAb is present in the solution, the $r_{h}$-value of HB-IgG-A in the buffer system (reference sample; pH 7) is measured to be 6.0 ± 0.4 nm (see black dashed line in Figure 2B). The results reveal that $r_{h}$-values increase with increasing Kleptose^®^HPB concentration (see Figure 2B). $r_{h}$-values of unfiltered and filtered samples at $c_{Kleptose^{®}HPB}$ = 250 mM differ significantly (see blank and filled squares in Figure 2B), again indicating the appearance of larger aggregates at these concentrations. However, measurements do not indicate an aggregation behavior of HB-IgG-A itself. As auto-association of Kleptose^®^HPB will typically produce aggregates < 2.2 nm, the increase in $r_{h}$-values can be attribute (at least in parts) to the attachment/close interaction of Kleptose^®^HPB to HB-IgG-A, further supported by the $M_{w}$-values determined for filtered HB-IgG-A-Kleptose^®^HPB systems (see Table A3 given in the Appendix A of the manuscript) revealing the increased attachment/close interaction of Kleptose^®^HPB aggregates with HB-IgG-A.

### 3.2. Surface Tension

As explained in the introduction, besides stabilization of the protein in solution, decreasing its surface activity is one major task of the excipients. Within this work, the surface tension σ of various aqueous Kleptose^®^HPB solutions were determined experimentally (see Section 2.2.2) to reveal if Kleptose^®^HPB does attach to the interface and potentially displace proteins from the interface (thus indirectly lowering their surface activity). Table 2 lists the σ-value of water, as well as the experimentally determined σ-values of aqueous HB-IgG-A (model protein) solution (pH 7) as a function of Kleptose^®^HPB. *ρ* of the respective systems (required for determination of σ—see Equation (1)) was determined by means of oscillating U-tube methods and the *ρ*-data are listed in Table A1 in Appendix A.

The results reveal that the surface tension σ of aqueous Kleptose^®^HPB solutions is lower compared to water/K_2_HPO_4_-NaH_2_PO_4_ solution and decreases as a function of Kleptose^®^HPB concentration. This indicates an increased surface activity of Kleptose^®^HPB. This effect is also shown for aqueous HB-IgG-A solution (55.15 mN m^−1^). However, the addition of Kleptose^®^HPB to this solution does (within the given accuracy) not indicate an increased surface activity of Kleptose^®^HPB.

### 3.3. Aggregate Formation

In order to identify the individual influence of Kleptose^®^HPB on (pre-existing) mAb aggregates, monomers as well as mAb fragments, samples containing HB-IgG-A in presence of Kleptose^®^HPB were analyzed using SEC-MALS as described in the materials and methods section (see Section 2.2.5).

**Figure 3:**
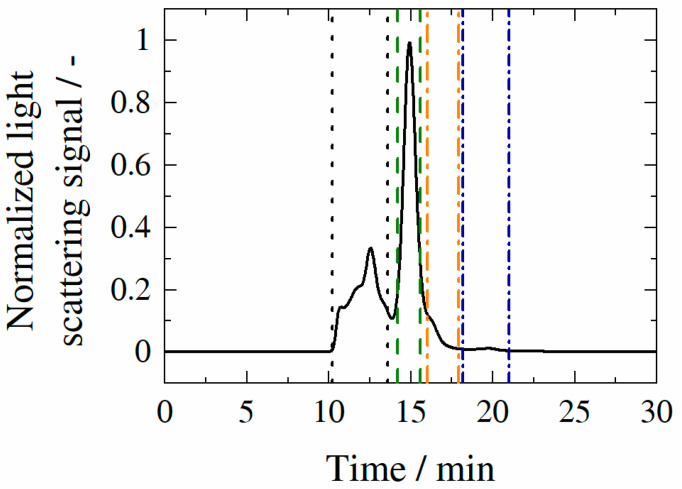
Experimentally determined chromatogram of HB-IgG-A in lean buffer (50 mM K_2_HPO_4_-NaH_2_PO_4_-solution; pH 7). The respective peaks can be assigned to the fractions within the sample: aggregates (framed by black dotted line); monomers (framed by green dashed line); fragments (framed by orange and blue dotted-dashed line).

Figure 3 illustrates exemplarily chromatogram resulting from SEC-MALS measurement of HB-IgG-A in lean buffer (50 mM K_2_HPO_4_-NaH_2_PO_4_-solution, pH 7).

The chromatogram of HB-IgG-A shows a discrete peak at a retention time of approximately 15 min, which can be assigned to the monomer of HB-IgG-A. However, the chromatogram of HB-IgG-A additionally reveals several peaks at lower retention times (10–14 min), which can be assigned to HB-IgG-A aggregates, and higher retention times (16–21 min), which can be assigned to HB-IgG-A fragments. In contrast to HB-IgG-A, samples containing HB-IgG-B have a comparable amount of aggregates (~20%) but drastically lower amount of fragments (~0.5%).

Table 3 lists the different fractions within aqueous HB-IgG-A and HB-IgG-B solutions in the presence of Kleptose^®^HPB (four different concentrations). The classification of the respective mass fraction is based on the differentiation made previously (see Figure 3).

Considering the mass distribution only, the results reveal that the addition of Kleptose^®^HPB to samples containing HB-IgG-A leads to a reduction in aggregate and monomer mass fraction as soon as the threshold value for Kleptose^®^HPB auto-association is exceeded (results at ≥ 150 mM Kleptose^®^HPB). The amount of the fragment fraction(s) of HB-IgG-A within those samples increases simultaneously. Table 4 lists the different molecular weights for the individual fractions (defined in Table 3) identified by static light scattering as described in the materials and methods Section 2.2.5 and Section 2.2.6. In contrast (to samples containing HB-IgG-A), when added to HB-IgG-B formulations, Kleptose^®^HPB does not affect aggregate, monomer, nor fragments fraction, irrespective of the concentration (of Kleptose^®^HPB) used. Moreover, no effect of increasing Kleptose^®^HPB concentration beyond the auto-association threshold is visible. Furthermore, non-specific hydrophilic binding of proteins to the column may be responsible for retention time shift and peak tailing, thus may affect the aggregation and fragmentation analysis.

The results reveal several interesting aspects on the phase behavior upon Kleptose^®^HPB addition. (1) For the addition of 5 and 10 mM Kleptose^®^HPB to aqueous HB-IgG-A solutions, the weight average molecular weight of the aggregates and monomer mass fraction does not change significantly. Only the weight average molecular weight of fragment 2 fractions decreases with increasing Kleptose^®^HPB concentration. (2) If the concentration threshold for Kleptose^®^HPB auto-association is exceeded (samples with 150 and 250 mM Kleptose^®^HPB—HB-IgG-A), and thus Kleptose^®^HPB aggregates are presents, the weight average molecular weight of the aggregate, monomer and fragments 1 fraction increases to a small extend, whilst the average molecular weight of fraction 2 decreases significantly. Taking into account, that the amount of fragment 2 fraction increases this indicates that fragment 2 fraction for these samples may contains a measurable amount of low-molecular weight components. These finding will be discussed in detail in Section 4.1.

### 3.4. Unfolding Temperature

**Figure 4:**
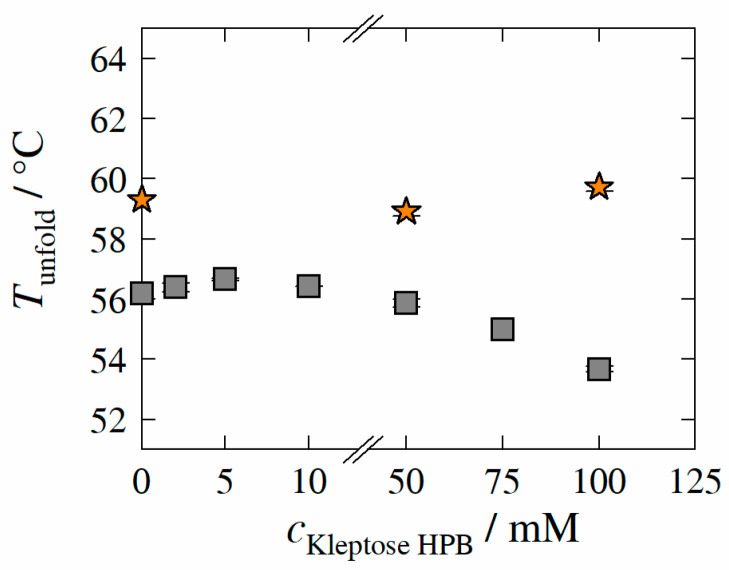
Experimentally determined unfolding temperature *T*_unfold_ of HB-IgG-A (gray square) and adalimumab (orange star) as function of Kleptose^®^HPB concentration. Data are valid for protein concentrations of 30 mg mL^−1^ (HB-IgG-A) or 8 g L^−1^ (adalimumab), pH 7 (HB-IgG-A) or pH 5.2 (adalimumab) in 50 mM K_2_HPO_4_-NaH_2_PO_4_-solution and a heating rate of 0.5 K min^−1^ in the temperature range of 20–85 °C.

In order to investigate the influence of Kleptose^®^HPB on the conformational mAb stability of HB-IgG-A and adalimumab, $T_{unfold}$ was measured as described in Section 2.2.3. Figure 4 illustrates $T_{unfold}$-values of HB-IgG-A (gray squares) or adalimumab (orange stars) as a function of Kleptose^®^HPB concentration.

$T_{unfold}$ of HB-IgG-A in the buffer system (reference sample; pH 7) was measured to 56.2 ± 0.1 °C. The results reveal that, $T_{unfold}$-values of HB-IgG-A are slightly affected by low Kleptose^®^HPB concentrations (2–10 mM), indicating increased/maximum conformational protein stability in the presence of 5 mM Kleptose^®^HPB. However, $T_{unfold}$-values of HB-IgG-A decreases linearly with increasing Kleptose^®^HPB concentrations ($c_{Kleptose^{®}HPB}$ > 50 mM), after exceeding the threshold concentration for Kleptose^®^HPB auto-association of 10 mM, indicating a concentration-dependent reduction in conformational protein stability.

$T_{unfold}$ of adalimumab in the buffer system (reference; pH 5.2) was measured to 59.3 ± 0.1 °C, indicating a higher initial conformational protein stability of adalimumab (compared to HB-IgG-A). In contrast to $T_{unfold}$-values of HB-IgG-A, the results reveal that $T_{unfold}$-values of adalimumab are not affected by any of the Kleptose^®^HPB concentrations considered within this work (50 and 100 mM), indicating constant conformational stability of adalimumab.

### 3.5. Aggregation Temperature

**Figure 5:**
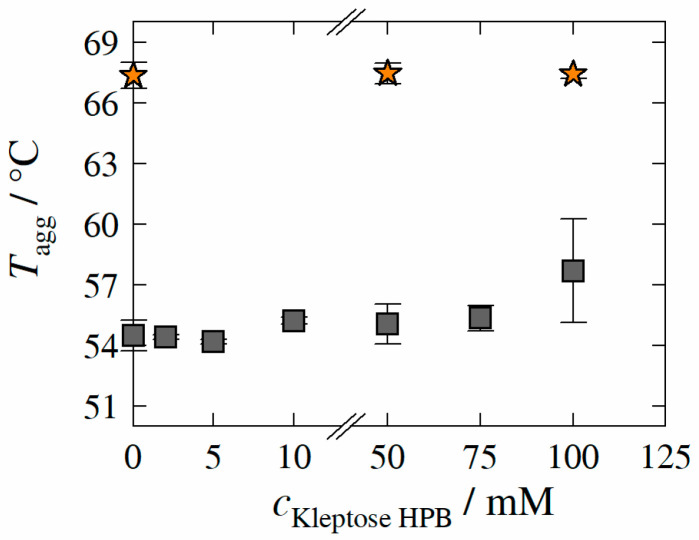
Experimentally determined aggregation temperature *T_agg_* of HB-IgG-A (gray square) and adalimumab (orange star) as function of Kleptose^®^HPB concentration. Data are valid for protein concentrations of 30 mg mL^−1^ (HB-IgG-A) or 10 mg mL^−1^ (adalimumab), pH 7 (HB-IgG-A) or pH 5.2 (adalimumab) in 50 mM K_2_HPO_4_-NaH_2_PO_4_-solution and a heating rate of 0.5 K min^−1^ in the temperature range of 20–85 °C.

In order to identify the influence of Kleptose^®^HPB on the colloidal stability of HB-IgG-A and adalimumab, $T_{agg}$-values of HB-IgG-A and adalimumab were determined experimentally as described in Section 2.2.3. Figure 5 illustrates $T_{agg}$-values of HB-IgG-A (gray squares) or adalimumab (orange stars) as a function of Kleptose^®^HPB concentration.

$T_{agg}$ of HB-IgG-A in the lean buffer system (reference, pH 7) was measured to 54.5 ± 0.8 °C. The results reveal that $T_{agg}$-values of HB-IgG-A (in contrast to $T_{unfold}$) remain constant over all Kleptose^®^HPB concentrations considered within this work (whether low (2–10 mM) or high (50 and 75 mM)), indicating constant aggregation propensity for HB-IgG-A. For the samples containing the highest Kleptose^®^HPB concentration (100 mM), the degree of uncertainty (large experimental error) does not allow for a clear conclusion.

$T_{agg}$ of adalimumab in lean buffer (reference; pH 5.2) was measured to 67.4 ± 0.6 °C, again indicating higher initial colloidal protein stability of adalimumab (compared to HB-IgG-A). As for HB-IgG-A systems, the results reveal that $T_{agg}$-values of adalimumab are not affected by any of the Kleptose^®^HPB concentrations considered (50 and 100 mM), indicating constant colloidal stability and aggregation propensity of adalimumab.

### 3.6. Protein–Protein Interactions

**Figure 6:**
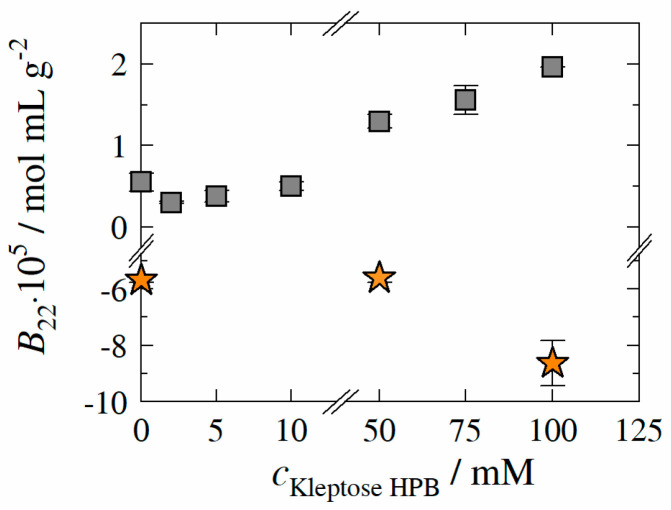
Experimentally determined second osmotic virial coefficient *B*_22_ of HB-IgG-A (gray square) and adalimumab (orange star) as function of Kleptose^®^HPB concentration. Data are valid for protein concentrations of 30 mg mL^−1^ (HB-IgG-A) or 10 mg mL^−1^ (adalimumab), 25 °C and pH 7 (HB-IgG-A) or pH 5.2 (adalimumab) in 50 mM K_2_HPO_4_-NaH_2_PO_4_ solution.

In order to provide a holistic insight into the molecular interactions, $B_{22}$-values of HB-IgG-A or adalimumab in the presence of different Kleptose^®^HPB concentrations were determined experimentally as described in Section 2.2.4. The second osmotic virial coefficient $B_{22}$ serves as a determinant of protein–protein interactions (PPI) and thus as indicator for colloidal mAb stability. Figure 6 illustrates the results of $B_{22}$-measurements of HB-IgG-A (gray squares) or adalimumab (orange stars) as a function of Kleptose^®^HPB concentration.

$B_{22}$ of HB-IgG-A in lean buffer (reference, pH 7) was measured to 0.55 ± 0.12∙10^−5^ mol mL g^−2^ indicating repulsive PPI. The results reveal that, $B_{22}$-values of HB-IgG-A are positive for all Kleptose^®^HPB concentrations investigated within this work, indicating net repulsive protein–protein interactions induced by Kleptose^®^HPB irrespective of its concentration. $B_{22}$-values of HB-IgG-A show a minimum at a Kleptose^®^HPB concentration of 2 mM, indicating an initial decrease of the net repulsive PPI/forces. Beyond this minimum (Kleptose^®^HPB concentration between 50–100 mM), $B_{22}$-value of HB-IgG-A increase with increasing Kleptose^®^HPB concentration (even beyond the $B_{22}$-value of HB-IgG-A in lean buffer). This indicates strengthening of the repulsive PPI and consequently reduction of aggregation propensity in the presence of high Kleptose^®^HPB concentrations. Worth mentioning the course of PPI (minima in PPI) is comparable to the course measured for $T_{unfold}$ (maximum in $T_{unfold}$) taking the concentration scale of Kleptose^®^HPB addition into account. In contrast, $B_{22}$-values of adalimumab are negative for both, lean buffer (reference, pH 5.2 − $B_{22}$ = −5.68 ± 0.09 × 10^−5^ mol mL g^−2^) and in presence of Kleptose^®^HPB, suggesting net attractive PPI. The results also reveal that $B_{22}$-values show a sharp decrease at a critical Kleptose^®^HPB concentration of $c_{Kleptose^{®}HPB}$ ≥ 50 mM, indicating highly attractive PPI in the presence of high Kleptose^®^HPB concentrations.

### 3.7. Formulation Stability (Adalimumab/HB-IgG-A) upon Storage at 4 °C

As described in the introduction, determination of mAb stability (stored and stressed) in the presence of Kleptose^®^HPB and combinations of other excipients, previously shown to deliver on promising formulation conditions (that is trehalose and l-arginine) is crucial for identifying the potential of Kleptose^®^HPB as (novel) “multirole“ excipient. Therefore, long-term stability samples containing HB-IgG-A or adalimumab were prepared in the respective aqueous base-buffer solution adding Kleptose^®^HPB (35 or 65 mM) alone, or in combination with l-arginine, as well as trehalose-l-arginine (concentrations of the respective excipients where chosen to deliver on beneficial solution conditions as identified within previous studies [45]). The different excipient compositions are listed in Table 5.

Long-term stability samples were prepared, stored, and measured as described in the materials and methods section (see Section 2.2.3, Section 2.2.5 and Section 2.2.6) The samples were analyzed with respect to aggregation temperature $T_{agg}$ of the respective mAb, aggregate-, monomer-fraction and fragments within the respective sample at the initial state (*t* = 0), after 4 weeks and after 24 weeks (HB-IgG-A) or 32 weeks (adalimumab).

**Figure 7:**
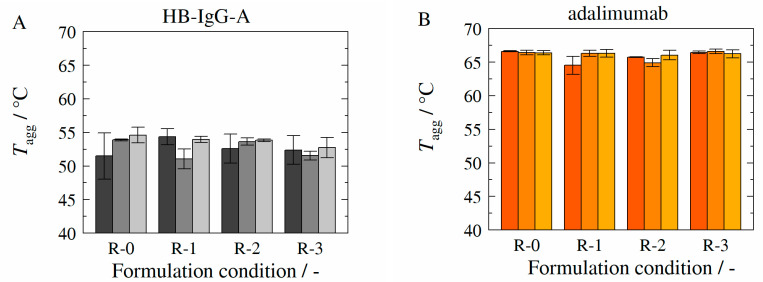
Experimentally determined aggregation temperature *T_agg_* in presence of HB-IgG-A (**A**) or adalimumab (**B**) at storage temperature of 4 °C as function of corresponding formulations (see Table 5) and storage duration. *T_agg_* of formulations containing HB-IgG-A (**A**) was measured at the initial state (dark gray bar), after 4 weeks (gray bar) and after 24 weeks (light gray bar). *T_agg_* of formulations containing adalimumab (**B**) was measured at the initial state (dark orange bar), after 4 weeks (orange bar) and after 32 weeks (light orange bar). Data are valid for protein concentrations of 85 g L^−1^ (HB-IgG-A) or 20 g L^−1^ (adalimumab), pH 7 (HB-IgG-A) or pH 5.2 (adalimumab) in 50 mM K_2_HPO_4_-NaH_2_PO_4_-solution and a heating rate of 0.5 K min^−1^ in the temperature range of 20–85 °C.

Figure 7 shows the experimentally determined aggregation temperatures $T_{agg}$ (see Section 2.2.3) of HB-IgG-A (A) and adalimumab (B) for the reference system (without excipients—formulation conditions R-0), for l-arginine-trehalose system (formulation conditions R-1), for l-arginine-Kleptose^®^HPB system (formulation conditions R-2) and for Kleptose^®^HPB system (formulation conditions R-3) at the initial state *t* = 0 (dark gray or orange bars), after 4 weeks (gray or orange bars) and after 24 weeks containing HB-IgG-A (light gray bars) or 32 weeks containing adalimumab (light orange bars). $T_{agg}$-values for both proteins (HB-IgG-A or adalimumab) do not vary significantly for the different samples and thus with respect to the formulation conditions investigated. However, $T_{agg}$-values for adalimumab are higher compared to those measured for HB-IgG-A, indicating/confirming the (initial) higher protein stability of adalimumab.

**Figure 8:**
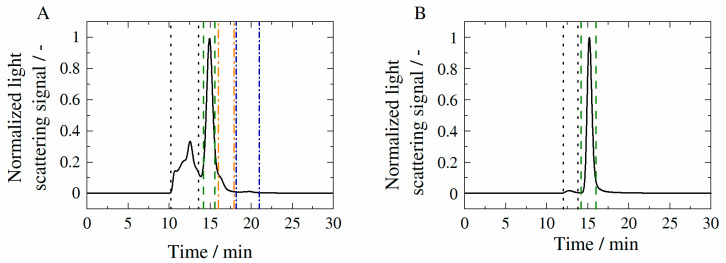
Chromatograms of HB-IgG-A (**A**) and of adalimumab (**B**) in 50 mM K_2_HPO_4_-NaH_2_PO_4_-solution at pH 7 (HB-IgG-A) or pH 5.2 (adalimumab). Based on the chromatograms, characteristic peaks were identified. The respective peaks can be assigned to the fractions within the sample: aggregates (framed by black dotted line); monomers (framed by green dashed line); fragments (framed by orange and blue dotted-dashed line).

Figure 8 illustrates exemplarily chromatograms resulting from SEC-MALS measurements (see Section 2.2.5) of the reference sample (formulation conditions R-0) with HB-IgG-A (A) or adalimumab (B) at the initial state (t=0).

The chromatograms of HB-IgG-A (see Figure 8A) and adalimumab (see Figure 8B) show a discrete peak at a retention time of approximately 15 min, which can be assigned to the respective monomer of each protein. Other fractions present in the case of HB-IgG-A have already been introduced in Section 3.3.

For systems containing adalimumab, the aggregate fraction (see retention times 10–13 min in Figure 8B) remained constant < 5% over the period considered (see Table A4 in the Appendix A of the manuscript) indicating suppression of aggregation by formulation conditions considered. In addition, the weight average molecular weight $M_{w}$ of each fraction is listed in Table A5 in the Appendix A of the manuscript.

For samples containing HB-IgG-A (without excipients–HB-IgG-A-R-0), in the presence of l-arginine-trehalose (HB-IgG-A-R-1), l-arginine-Kleptose^®^HPB (HB-IgG-A-R-2) and only Kleptose^®^HPB system (HB-IgG-A-R-3) results of respective mass fraction are shown in Table A6 in the Appendix A of the manuscript. The weight average molecular weight $M_{w}$ of each fraction is listed in Table A7 in the Appendix A of the manuscript.

The results reveal aggregate- and monomer mass fractions of HB-IgG-A within the sample decreases while fragment mass fractions increase over time for all formulation conditions considered. As expected, the decrease/increase is strongest for formulations without excipients (HB-IgG-A-R-0). It has to be noted that due to the initial instability/large aggregate fraction already included in the HB-IgG-A samples, long-term stability experiments at 4 °C do not allow for a valid conclusion on the effectiveness of the excipients considered by means of stabilization.

### 3.8. Formulation Stability (Adalimumab/HB-IgG-A) upon Storage at 40 °C

In order to evaluate the effectiveness of the excipients considered toward stabilization of the respective mAbs in solution, samples (adalimumab and samples of HB-IgG-A including Kleptose^®^HPB) were exposed to 40 °C storage conditions (stored in a ThermoMixer of Eppendorf, Hamburg, Germany) to allow for stability tests against thermal induced stress at early stages.

**Figure 9:**
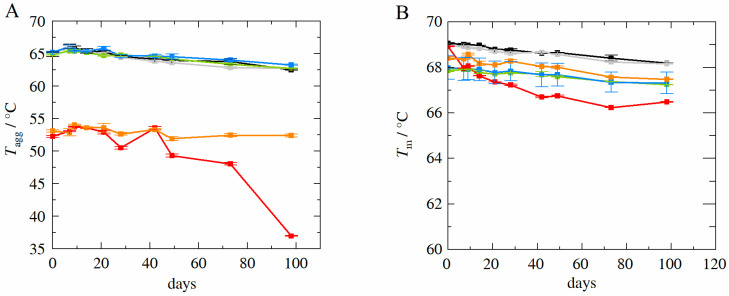
(**A**): Experimentally determined aggregation temperature *T_agg_* for HB-IgG-A and adalimumab at storage temperature of 40 °C as function of storage duration. (**B**): Experimentally determined melting temperature *T_m_* for HB-IgG-A and adalimumab at storage temperature of 40 °C as function of storage duration. Data are valid for protein concentrations of 85 g L^−1^ (HB-IgG-A) or 20 g L^−1^ (adalimumab), pH 7 (HB-IgG-A) or pH 5.2 (adalimumab) in 50 mM K_2_HPO_4_-NaH_2_PO_4_-solution and a heating rate of 0.5 K min^−1^ in the temperature range of 20–85 °C. Formulations containing adalimumab: adalimumab-R-0—black, adalimumab-R-1—grey, adalimumab-R-2—green, adalimumab-R-3—blue; formulations containing HB-IgG-A: HB-IgG-A-R-2—red, HB-IgG-A-R-3—orange.

Figure 9A shows the experimentally determined aggregation temperatures $T_{agg}$ as a function of storage days for long-term samples stored at 40 °C in the temperature controlled ThermoMixer (accelerated studies). Samples contained HB-IgG-A for l-arginine-Kleptose^®^HPB system (HB-IgG-A-R-2—red line) and for Kleptose^®^HPB system (HB-IgG-A-R-3—orange line), as well as for adalimumab in the reference system (adalimumab-R-0—black), l-arginine-trehalose system (adalimumab-R-1—grey), l-arginine-Kleptose^®^HPB system (adalimumab-R-2—green), and Kleptose^®^HPB system (adalimumab-R-3—blue).

With the exemption of the HB-IgG-A-R-2 containing l-arginine-Kleptose^®^HPB, $T_{agg}$ values do not vary significantly within the duration considered (both for HB-IgG-A as well as adalimumab), showing a slow decrease in $T_{agg}$.(~2 °C over 100 days). $T_{agg}$ values for systems containing adalimumab are higher compared to those measured for HB-IgG-A, as already shown for long-term samples stored at 4 °C, indicating higher stability of adalimumab.

Figure 9B shows the experimentally determined melting temperatures $T_{m}$ as a function of storage days for long-term samples stored at 40 °C (accelerated studies). Samples contained HB-IgG-A for l-arginine-Kleptose^®^HPB system (HB-IgG-A-R-2—red line) and for Kleptose^®^HPB system (HB-IgG-A-R-3—orange line), as well as for adalimumab in the reference system (adalimumab-R-0—black line), l-arginine-trehalose system (adalimumab-R-1—grey line), l-arginine-Kleptose^®^HPB system (adalimumab-R-2—green), and Kleptose^®^HPB system (adalimumab-R-3—blue line).

Again, with the exemption of the HB-IgG-A-R-2 containing l-arginine-Kleptose^®^HPB, $T_{m}$-values do not vary significantly within the duration considered (both for HB-IgG-A as well as adalimumab), showing a slow decrease also in $T_{m}$.(~1.5 °C over 100 days). Measurements with respect to the monomer mass recovery (SEC-MALS measurements as described in the materials and methods section) reveal that HB-IgG-A samples precipitated to a significant extent (less than 10% monomer recovery) after 100 days of storage at 40 °C. However, as the sample initially already included a significant amount of fragments and aggregates, it is plausible that this initial (in)stability was the cause for the increased (thermal) instability at 40 °C.

**Figure 10:**
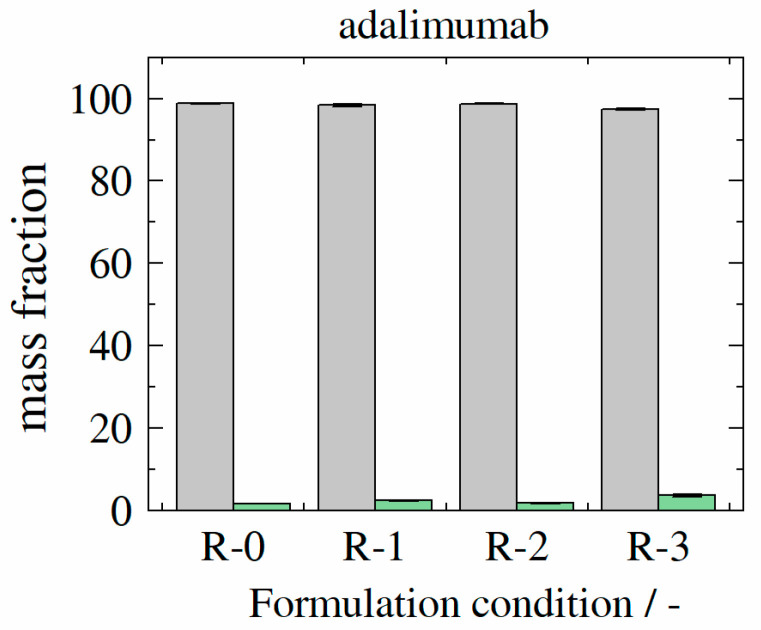
Experimentally determined monomer (gray bars) and aggregate (green bars) mass fractions for formulations (formulation conditions R-0–R-3) containing adalimumab at storage temperature of 40 °C as after 100 days.

In contrast, adalimumab samples revealed a high monomer mass recovery of at least 97.4% for adalimumab sample adalimumab-R-3, 98.8% for sample adalimumab-R-2, 98.1% for sample adalimumab-R-1, and 98.2% for sample adalimumab-R-0 (see Figure 10).

## 4. Discussion

### 4.1. Auto-Association and Aggregation Propensity of Kleptose^®^HPB

The results from Section 3.1 reveal that the onset of auto-association already occurs at rather low Kleptose^®^HPB concentrations (2–10 mM), identifiable by the increase of the molecule size ($r_{h}$). An effect of the buffer components (e.g., salts) can be excluded, since the investigations for Kleptose^®^HPB in lean water led to the same results (not included). The auto-association can be attributed to the molecular structure and especially to the hydroxypropyl groups of Kleptose^®^HPB, which leads to attractive interactions due to the formation of hydrogen bonds. This hypothesis is supported by the concentration-dependent increase in viscosity for aqueous Kleptose^®^HPB solutions (data not included) and previous findings of Häusler et al. [7] and Gokhale [17] who identify the formation of hydrogen bonds as the cause for the auto-association of HP-β-CD (that is Kleptose^®^HPB) by viscosity- and differential scanning calorimetry measurements. However, the results (see Section 3.1) also reveal that with increasing Kleptose^®^HPB concentration (50–250 mM) the auto-association propensity increases, and the formation of larger aggregates is preferred. This evidence is not only supported by the increase in $r_{h}$-values, but also by the difference between $r_{h}$-values of filtered and non-filtered samples. These findings are in good agreement with studies made by Sá Couto et al. [21] who identified an increased aggregation propensity of HP-β-CD with increasing concentration using DLS- and filtration methods. Furthermore, the $r_{h}$-values suggest a critical aggregation concentration (cac) between 10 and 50 mM, which is in good agreement with cac identified by Sá Couto et al. [21] and Do et al. [49] for different types of HP-β-CD in the range of 2–12% *m/v* using DLS and H’-NMR analytics.

### 4.2. Surface Activity Induced by Kleptose^®^HPB

The results of Section 3.2 revealed that Kleptose^®^HPB leads to a decrease in surface tension already at very low concentrations (below cac; 5 and 10 mM). These results are in good agreement with previous studies made by Sá Couto et al. [21] and Loftsson [22] who identified a concentration-dependent influence of HP-β-CD on surface tension reduction. However, an increased displacement of HB-IgG-A could not be observed, as HB-IgG-A itself already leads to a strong reduction of surface tension (see Table 2). It is unlikely that HP-β-CD can replace classical surfactants such as polysorbate 80 [18,24].

### 4.3. Interaction Characteristics between Kleptose^®^HPB and Proteins (HB-IgG-A & Adalimumab)

Building up on the auto-association and aggregation propensity of Kleptose^®^HPB, we propose that the impact of Kleptose^®^HPB on protein aggregation propensity (as well as on interaction characteristics between Kleptose^®^HPB and proteins) depends on at least three factors:

- Kleptose^®^HPB concentration (bellow/above cac);
- Protein type and protein concentration;
- Quality of protein in solution (aggregate fraction/amount & fragment fraction).

The results on conformational and colloidal mAb stability ($T_{unfold}$, $T_{agg}$, $B_{22}$) clearly indicate that interaction characteristics of Kleptose^®^HPB strongly depend on the state of the mAb sample. If the sample/formulation is pure (meaning high monomeric content) as for the adalimumab or HB-IgG-B, Kleptose^®^HPB does not have any decisive influence (negative or positive) at low Kleptose^®^HPB concentrations. As soon as the threshold of Kleptose^®^HPB auto-association is exceeded, $T_{unfold}$ and $T_{agg}$ still do not show a clear trend, but $B_{22}$ measurements reveal net attractive interactions of the mAb in the presence of Kleptose^®^HPB. This statement is supported by all measurements on conformational and colloidal mAb stability with respect to adalimumab samples.

If the mAb sample is impure (aggregates and fragments already present in solution) concentration-dependent measurements using Kleptose^®^HPB do reveal interaction mechanisms that can take place.

As seen in the results for the SEC fractionated samples for HB-IgG-A (impure-significant fragment fraction) and HB-IgG-B (low fragment fraction), Kleptose^®^HPB at low concentrations of 5 and 10 mM does not show any decisive interaction with any of the protein fractions considered. At higher Kleptose^®^HPB concentrations (>50 mM) the interaction scheme is inconclusive. Samples containing HB-IgG-A show that Kleptose^®^HPB at least partially interacts with the protein. The increase in weight average molecular weight of aggregate and monomer fraction indicates that either single Kleptose^®^HPB molecules attach to the mAb (most likely true for the monomer), or large Kleptose^®^HPB clusters interact or attach to the monomers and aggregates fraction. With respect to fragment fraction 2, the weight average molecular weight decreases whilst the amount of the fraction increases. This could indicate that besides HB-IgG-A fragments, also cluster of small molecular weight components (which may or may not interact with the fragments) are detected, increasing their amount but lowering the apparent weight average molecular weight.

**Figure A2:**
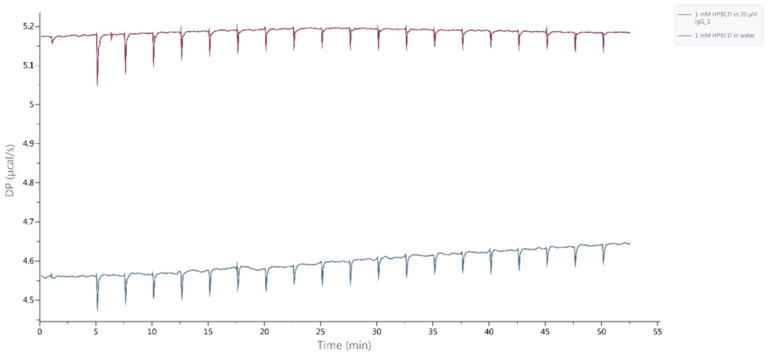
Isothermal titration calorimetry (ITC) measurements for HB-IgG-B. Blue curve: Titrating 1 mM Kleptose^®^HPB to 20 µM HB-IgG-B in buffer. Magenta: Titrating 1 mM Kleptose^®^HPB to buffer.

**Figure A3:**
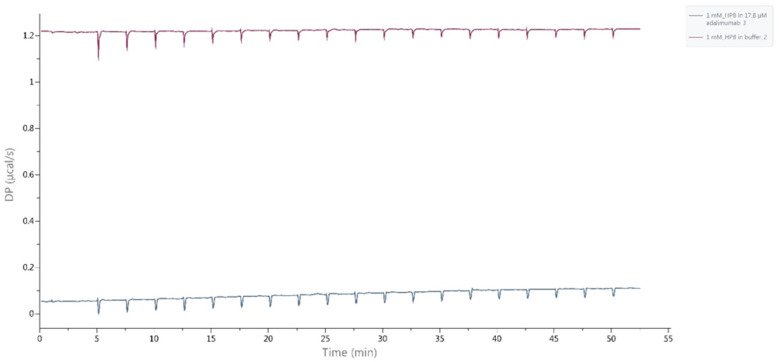
Isothermal titration calorimetry (ITC) measurements for adalimumab. Blue curve: Titrating 1 mM Kleptose^®^HPB to 17.8 µM adalimumab in buffer. Magenta: Titrating 1 mM Kleptose^®^HPB to buffer.

This hypothesis is supported by measurements with respect to $T_{unfold}$ where initially we see an increase in conformational stability for HB-IgG-A at low Kleptose^®^HPB concentrations < 50 mM followed by a decrease in conformational stability when exceeding these concentrations. PPI measurements underline these assumptions, as for low Kleptose^®^HPB concentrations we do see a reduction in net repulsive PPI up to a Kleptose^®^HPB concentration of ≤50 mM, due to the possible increasing attachment of Kleptose^®^HPB to fragments at these concentrations. At high Kleptose^®^HPB concentrations, net repulsive PPI increases due to the now possible dominant interactions of Kleptose^®^HPB aggregates with the HB-IgG-A monomers and aggregates. In contrast, results obtained for HB-IgG-B reveal that especially the presence of impurities such as a high fragment content is crucial in terms of apparent interactions in solution. For the low fragment content in these samples, no negative effect could be determined for any of the fractions considered. In combination with the previous results, it is likely that the presence of the fragment fraction rather than high Kleptose^®^HPB concentrations is responsible for the instability determined for HB-IgG-A samples. These finding are supported by ITC measurements (see Figure A2 and Figure A3 given in the Appendix A of the manuscript) revealing no interaction between Kleptose^®^HPB and HB-IgG-B at low Kleptose^®^HPB concentrations. Again, it has to be mentioned that these results are specific for the proteins considered within this work and need to be confirmed for other proteins and protein concentrations.

### 4.4. Potential of Kleptose^®^HPB as Excipient (Long-Term Stability Studies)

Based on the results of the long-term stability studies ($T_{agg}$-measurements and mass fraction analysis) at storage conditions of 4 °C, the changes in mAb long-term stability/mass fraction are mainly due to the initial quality/instability of the mAb, but not to the interaction of excipients with the mAb itself. Formulation conditions R-2 (Kleptose^®^HPB-l-arginine) and formulation conditions R-3 (Kleptose^®^HPB) contain Kleptose^®^HPB concentrations above cac/threshold of Kleptose^®^HPB auto-association (see Section 4.1.), so auto-association of Kleptose^®^HPB occurs within these formulations. However, the constant $T_{agg}$-values reveal that this auto-association does not affect the colloidal stability of the mAb, either for HB-IgG-A or adalimumab. Furthermore, constant monomer/aggregate mass fractions of adalimumab over time for all formulation conditions considered reveal that both the presence of these Kleptose^®^HPB aggregates and trehalose-l-arginine does not affect the adalimumab stability/aggregation propensity due to the initial high stability of adalimumab.

The results of the long-term stability studies ($T_{agg}$ and $T_{m}$—measurements and mass fraction analysis) at storage conditions of 40 °C primarily provide an indication of excipient influence on mAb stability under thermally induced stress and can only be used to a limited extent as a criterion for the long-term stability of mAbs within the formulation. Nevertheless, the constant $T_{agg}$ and $T_{m}$-values of the accelerated studies show that Kleptose^®^HPB in mixtures with l-arginine (see formulation adalimumab-R-2 in Figure 9B) or alone might serve as a stabilizer for thermally induced stress. HB-IgG-A were not meaningful in this regard, as almost all monomer precipitated.

Suvarna et al. [50] and Sherje et al. [51] identified the formation of HP-β-CD-l-arginine complexes within ternary complex formation with small API (such as Nateglinide and Zaltoprofen) via nuclear magnetic resonance (^1^HNMR). The complex formation of HP-β-CD-l-arginine is attributed to the interaction of the hydrophobic region of l-arginine and hydrophobic region of HP-β-CD (resulting in hydrogen bonding, electrostatic interaction and salt formation) [50,51]. The complex formation, which is also suggested for Kleptose^®^HPB-l-arginine, could consequently induce the stabilization of the mAb against thermally induced stress. However, based on our accelerated long-term stability studies alone, we cannot conclusively clarify the extent to which this complex interacts with the mAb. The stabilization of the mAb could be caused both by the direct interaction between the complex formed and the mAb (e.g., binding, hydrogen bonds) and by strong water-excipient (here: complex formed) interactions which result in “excluded volume” mechanisms known from sugars such as trehalose and sucrose [52,53,54] (mAb is surrounded by aqueous layer, which is not penetrate by the complex formed).

## 5. Conclusions

Within this work we investigated the interactions and interaction mechanisms of Kleptose^®^HPB used as an excipient within different HCPFs (HB-IgG, adalimumab as mAb).

Besides validating the auto-association (above cac) of Kleptose^®^HPB and its influence on surface activity, three major factors were identified that determine whether Kleptose^®^HPB has a significant impact on mAb interactions in solution: (1) concentration of Kleptose^®^HPB (bellow/above cac), (2) protein type and protein concentration, and (3) quality of the mAb formulation (aggregate/fragment mass fractions of mAb). For high-quality samples investigated within this work (high monomer/low aggregate fraction or high monomer/low fragment fraction), as here for adalimumab and HB-IgG-B, the conformal/colloidal mAb stability (constant $T_{unfold}$-/$T_{agg}$-values) was not affected by Kleptose^®^HPB (independent of concentration and appearance of Kleptose^®^HPB auto-association). Its plausible that Kleptose^®^HPB assists in stabilizing the mAb in solution, although these results are strongly dependent on antibody type and concentration considered.

This is confirmed, as for low-quality samples (high aggregate/low monomer /high fragment mass fraction of mAb), as here for HB-IgG-A, the interaction mechanisms identified depends strongly on the Kleptose^®^HPB concentration (bellow/above cac), as well as the individual fraction of the antibody considered (aggregates, monomers, fragments). If the sample already shows a high initial (in)stability, the use of Kleptose^®^HPB at high concentrations might not be beneficial. However, our results for adalimumab indicate that the use of Kleptose^®^HPB with l-arginine can be a promising excipient mixture for exploiting synergetic mAb stabilization effects (constant $T_{agg}$-values) through Kleptose^®^HPB-l-arginine complex formation.

Based on our results, we were able to gain a comprehensive insight into the prevailing interaction characteristics between Kleptose^®^HPB and two mAbs. Furthermore, we identified the Kleptose^®^HPB application potential in HCPFs. These results further highlight the particular need for identification of the interaction/complexation of Kleptose^®^HPB with other proteins and other excipients and applications to further exploit the potential.

In general, our studies close a gap in the knowledge and understanding of the interaction characteristics between Kleptose^®^HPB and mAb which enable a more targeted use of Kleptose^®^HPB as an excipient within the design of liquid protein (mAb) formulations.

## Figures and Tables

**Table 1:** List of chemicals used and the respective chemical abstract service (CAS) number, formula, purity and supplier.

Component	CAS-No	Formula	Purity	Supplier
HB-IgG-A	9007-83-4	-	≥99.0%	Sigma Aldrich, Schnelldorf, Germany
HB-IgG-B	NA (LOT#BS19201051)	-	98.0%	BOC Science, Shirley, NY, USA
Adalimumab-solution	-	-	-	In-house preparation Roquette Asia Pacific Pte. Ltd., Singapore
Kleptose^®^HPB	128446-35-5		≥97.0%	Roquette Freres, Lestrem, France
l-arginine HCl	1119-34-2	C_6_H_14_N_4_O_2_·HCl	≥98.0%	Sigma Aldrich, Schnelldorf, Germany
Dipotassium hydrogen phosphate	7758-11-4	K_2_HPO_4_	≥99.8%	BDH Prolabo, VWR, Langenfeld, Germany
Sodium dihydrogen phosphate	7558-80-7	NaH_2_PO_4_	≥98.0%	BDH Prolabo, VWR, Langenfeld, Germany

**Table 2:** Experimentally determined surface tension σ of aqueous HB-IgG-A K_2_HPO_4_-NaH_2_PO_4_ solution as function of Kleptose^®^HPB (pH 7). The measurement was performed at 25 °C and a pressure of 1 bar. The corresponding density data (necessary for *σ*-determination) are listed in Table A1 given in the Appendix A of the manuscript.

System	$c_{Kleptose^{®}HPB}$ /mM	$c_{HB-IgG-A}$ /mg mL^−1^	σ /mN m^−1^
Water	-	-	71.97 [28]
Kleptose^®^HPB in K_2_HPO_4_-NaH_2_PO_4_ solution	5.1 ± 0.3	-	61.49 ± 0.23
	9.8 ± 0.1	-	60.27 ± 0.09
HB-IgG-A in K_2_HPO_4_-NaH_2_PO_4_ solution	-	30.0 ± 0.4	55.15 ± 0.01
HB-IgG-A + Kleptose^®^HPB in K_2_HPO_4_-NaH_2_PO_4_ solution	4.9 ± 0.2	29.8 ± 0.3	54.86 ± 0.08
	9.9 ± 0.3	30.4 ± 0.3	54.71 ± 0.09

**Table 3:** Experimentally determined aggregate, monomer and fragment mass fractions within aqueous HB-IgG-A and HB-IgG-B solutions (quality difference see materials and methods) in the presence of Kleptose^®^HPB.

System	Aggregates /%	Monomer /%	Fragment (1) /%	Fragment (2) /%
HB-IgG-A	20.3 ± 0.4	68.0 ± 0.4	5.8 ± 0.1	2.7 ± 0.1
HB-IgG-A + 5 mM Kleptose^®^HPB	20.2 ± 0.1	67.9 ± 1.1	5.7 ± 0.1	2.5 ± 0.6
HB-IgG-A + 10 mM Kleptose^®^HPB	20.9 ± 0.1	66.7 ± 0.1	5.7 ± 0.1	2.8 ± 0.1
HB-IgG-A + 150 mM Kleptose^®^HPB	18.8 ± 0.2	64.8 ± 0.2	4.9 ± 0.4	7.0 ± 0.5
HB-IgG-A + 250 mM Kleptose^®^HPB	17.5 ± 0.1	65.2 ± 0.3	5.1 ± 0.1	7.7 ± 0.1
HB-IgG-B	22.8 ± 0.03	76.1 ± 0.02	0.46 ± 0.005	0.58 ± 0.005
HB-IgG-B + 5 mM Kleptose^®^HPB	22.7 ± 0.07	76.3 ± 0.1	0.43 ± 0.02	0.56 ± 0.005
HB-IgG-B + 10 mM Kleptose^®^HPB	22.5 ± 0.03	76.5 ± 0.04	0.44 ± 0.025	0.57 ± 0.015
HB-IgG-B + 150 mM Kleptose^®^HPB	22.1 ± 0.03	76.8 ± 0.02	0.43 ± 0.025	0.58 ± 0.015
HB-IgG-B + 250 mM Kleptose^®^HPB	22.4 ± 0.11	76.58 ± 0.09	0.41 ± 0.015	0.55 ± 0.01

**Table 4:** Experimentally determined weight average molecular weight *M*_w_ of aggregate, monomer and fragment fractions within aqueous HB-IgG-A solutions in the presence of Kleptose^®^HPB.

System	Aggregates /kDa	Monomer /kDa	Fragments (1) /kDa	Fragments (2) /kDa
HB-IgG-A in lean buffer	343.7 ± 1.6	148.9 ± 0.3	119.8 ± 0.2	80.5 ± 5.3
HB-IgG-A + 5 mM Kleptose^®^HPB	351.5 ± 11.2	148.9 ± 0.9	118.6 ± 4.0	77.9 ± 18.2
HB-IgG-A + 10 mM Kleptose^®^HPB	345.2 ± 1.3	148.2 ± 0.2	116.1 ± 0.2	65.6 ± 2.8
HB-IgG-A + 150 mM Kleptose^®^HPB	398.6 ± 1.9	153.5 ± 0.1	144.0 ± 0.5	39.6 ± 0.2
HB-IgG-A + 250 mM Kleptose^®^HPB	377.1 ± 1.8	152.8 ± 0.3	138.2 ± 2.1	43.0 ± 4.0

**Table 5:** Excipient compositions of four formulations used for long term stability studies.

Formulation Condition	${\tilde{m}}_{Kleptose^{®}HPB}$ /mmol kg^−1^	${\tilde{m}}_{trehalose}$ /mmol kg^−1^	${\tilde{m}}_{L-arginine}$ /mmol kg^−1^
R-0	0	0	0
R-1	0	55	50
R-2	55	0	50
R-3	100	0	0

## Appendix A

**Table A1:** Experimentally determined densities of HB-IgG-A in 50 mM K_2_HPO_4_-NaH_2_PO_4_ buffer solution and in presence of Kleptose^®^HPB at 25 °C.

System	$c_{Kleptose^{®}HPB}$ /mM	$c_{HB-IgG}$ /mg mL^−1^	ρ (25 °C) /kg m^−3^
A	0	30.0 ± 0.4	1010.6 ± 0.2
B	4.9 ± 0.2	29.8 ± 0.3	1012.4 ± 0.6
C	9.9 ± 0.3	30.4 ± 0.3	1014.6 ± 0.5
D	5.1 ± 0.3	-	1011.2 ± 0.6
E	9.8 ± 0.1	-	1011.3 ± 0.5

**Table A2:** Experimentally determined viscosity of the aqueous Kleptose^®^HPB solution at 25 °C.

${\tilde{m}}_{Kleptose^{®}HPB}$ /mmol kg^−1^	η (25 °C) /mPa s
0.0542	1.1446 ± 0.0004
0.1150	1.5066 ± 0.0007
0.1796	2.0760 ± 0.0001
0.2556	2.9833 ± 0.0008
0.3381	4.4709 ± 0.0011

The particle size distributions illustrated in Figure A1A show that the unfiltered samples of the aqueous Kleptose^®^HPB solutions have particle size distributions in the range of $r_{h}$ of 0.8–1.2 nm. For aqueous Kleptose^®^HPB solutions with $c_{Kleptose^{®}HPB}$ > 5 mM, an additional particle size distribution in the range of 100 nm can be identified (see blue, red, green and gray lines in Figure A1A). For aqueous Kleptose^®^HPB solutions with $c_{Kleptose^{®}HPB}$ between 100 and 250 mM the unfiltered samples have additional particle size distributions in the range of high $r_{h}$-values between 1000 and 10,000 nm.

**Table A3:** Experimentally determined (via DLS) weight average molecular weight *M*_w_ of unfiltered and filtered (PES syringe filter with a pore size of 0.2 μm) samples as function of Kleptose^®^HPB and HB-IgG-A (30 mg mL^−1^) in 50 mM K_2_HPO_4_-NaH_2_PO_4_ solution at 25 °C and pH 7.

System	$c_{Kleptose^{®}HPB}$ /mM	$c_{HB-IgG}$ /mg mL^−1^	$M_{w}$ /Da
unfiltered	50	-	1452.4 ± 4.3
	150	-	1373.7 ± 8.6
	250	-	1488.1 ± 148.7
filtered	50	-	1443.2 ± 42.4
	150	-	1055.6 ± 3.7
	250	-	927.7 ± 13.3
unfiltered	50	30	158,402.5 ± 2233.8
	150	30	150,824.2 ± 2517.1
	250	30	163,823.2 ± 12,474.5
filtered	50	30	155,512.7 ± 105.7
	150	30	134,833.6 ± 204.0
	250	30	135,965.1 ± 90.3

**Table A4:** Experimentally determined mass fractions within long-term samples containing adalimumab at initial state (0 weeks), after 4 weeks, and after 32 weeks. The fractions result from subdivision according to chromatogram peaks (see Figure 8B).

		Mass Fraction/%			
		Adalimumab-			
	Weeks	R-0	R-1	R-2	R-3
Aggregates	0	0.10 ± 0.02	0.60 ± 0.28	0.30 ± 0.09	0.45 ± 0.07
	4	0.65 ± 0.21	0.90 ± 0.14	0.75 ± 0.07	0.80 ± 0.28
	32	1.25 ± 0.22	1.20 ± 0.24	1.45 ± 0.49	1.20 ± 0.71
Monomer	0	99.05 ± 1.06	98.85 ± 0.64	99.40 ± 0.71	98.85 ± 0.21
	4	98.30 ± 0.14	97.95 ± 0.35	98.20 ± 0.28	98.10 ± 0.57
	32	97.90 ± 0.28	97.80 ± 0.24	97.35 ± 0.78	97.8 ± 0.85

**Table A5:** Experimentally determined weight average molecular weight *M_w_* of respective fractions within long-term samples containing adalimumab at initial state (0 weeks), after 4 weeks, and after 32 weeks. The respective fractions results from subdivision according to chromatogram peaks (see Figure 8B).

		$M_{w}$/kDa			
		Adalimumab-			
	Weeks	R-0	R-1	R-2	R-3
Aggregates	0	587.5 ± 4.8	443.6 ± 84.3	508.4 ± 5.3	464.0 ± 6.8
	4	453.6 ± 45.8	391.7 ± 3.3	422.2 ± 23.7	404.4 ± 39.0
	32	537.8 ± 2.3	666.8 ± 43.0	468.2 ± 8.3	655.9 ± 71.2
Monomer	0	148.8 ± 0.1	147.8 ± 0.1	147.6 ± 0.1	147.4 ± 0.1
	4	147.5 ± 0.3	146.7 ± 0.3	146.8 ± 0.2	146.6 ± 0.3
	32	151.7 ± 0.8	150.9 ± 1.1	149.85 ± 1.8	151.3 ± 0.4

**Table A6:** Experimentally determined mass fractions within long-term samples containing HB-IgG-A at initial state (0 weeks), after 4 weeks, and after 24 weeks. The fractions results from subdivision according to chromatogram peaks (see Figure 8B).

		Mass Fraction/%			
		HB-IgG-A			
	Weeks	R-0	R-1	R-2	R-3
Aggregates	0	25.35 ± 0.92	22.70 ± 0.14	21.85 ± 0.21	21.90 ± 0.14
	4	23.45 ± 0.09	20.95 ± 0.07	21.55 ± 0.22	20.50 ± 0.28
	24	18.05 ± 0.21	20.30 ± 0.24	16.20 ± 0.09	18.45 ± 0.21
Monomer	0	61.30 ± 0.14	64.10 ± 0.28	65.15 ± 0.07	64.60 ± 0.99
	4	54.95 ± 2.83	62.75 ± 0.17	65.60 ± 4.67	63.20 ± 0.28
	24	41.75 ± 0.08	53.20 ± 0.28	50.70 ± 0.28	53.15 ± 0.17
Fragment (1)	0	7.90 ± 0.28	7.50 ± 0.14	7.60 ± 0.14	7.50 ± 0.14
	4	10.30 ± 0.42	9.20 ± 0.15	9.30 ± 0.13	9.40 ± 0.13
	24	14.65 ± 0.07	12.50 ± 0.23	14.20 ± 0.17	13.15 ± 0.07
Fragment (2)	0	2.75 ± 0.92	2.85 ± 0.21	2.70 ± 0.13	3.15 ± 0.49
	4	6.15 ± 1.48	4.10 ± 0.16	2.80 ± 2.26	3.95 ± 0.11
	24	20.95 ± 0.07	9.75 ± 0.21	14.55 ± 0.21	11.05 ± 0.07

**Table A7:** Experimentally determined weight average molecular weight *M*_w_ of respective fractions within long-term samples containing HB-IgG-A at initial state (0 weeks), after 4 weeks, and after 24 weeks. The respective fractions results from subdivision according to chromatogram peaks (see Figure 8A).

		$M_{w}$/kDa			
		HB-IgG-A			
	Weeks	R-0	R-1	R-2	R-3
Aggregates	0	399.2 ± 4.2	386.5 ± 3.3	390.6 ± 7.5	384.3 ± 4.1
	4	378.1 ± 8.3	386.9 ± 2.8	374.6 ± 22.8	374.4 ± 1.6
	24	419.5 ± 1.27	413.5 ± 12.3	381.9 ± 2.3	387.5 ± 1.2
Monomer	0	152.1 ± 0.8	151.5 ± 1.2	151.8 ± 0.2	151.8 ± 0.1
	4	156.2 ± 2.8	153.0 ± 1.1	152.9 ± 3.9	152.5 ± 0.5
	24	151.0 ± 0.5	150.8 ± 0.1	151.6 ± 0.2	150.7 ± 0.2
Fragments (1)	0	124.6 ± 3.3	127.7 ± 0.1	127.5 ± 1.0	128.1 ± 2.7
	4	125.3 ± 6.2	126.2 ± 0.1	121.2 ± 0.8	122.6 ± 1.8
	24	111.2 ± 0.5	114.7 ± 0.7	113.9 ± 1.0	114.6 ± 1.3
Fragments (2)	0	117.1 ± 47.8	99.0 ± 0.6	103.9 ± 2.1	86.2 ± 9.0
	4	84.0 ± 35.2	86.4 ± 4.3	88.1 ± 1.6	86.0 ± 1.3
	24	54.35 ± 0.8	59.1 ± 1.0	56.6 ± 2.1	58.8 ± 2.1
